# *Streptococcus mutans*, *Candida albicans*, and the Human Mouth: A Sticky Situation

**DOI:** 10.1371/journal.ppat.1003616

**Published:** 2013-10-17

**Authors:** Khalid H. Metwalli, Shariq A. Khan, Bastiaan P. Krom, Mary Ann Jabra-Rizk

**Affiliations:** 1 Department of Oncology and Diagnostic Sciences, Dental School, University of Maryland, Baltimore, Maryland, United States of America; 2 Department of Preventive Dentistry, Academic Centre for Dentistry Amsterdam (ACTA), University of Amsterdam and Free University Amsterdam, Amsterdam, The Netherlands; 3 Department of Microbiology and Immunology and Department of Pathology, School of Medicine, University of Maryland, Baltimore, Maryland, United States of America; Duke University Medical Center, United States of America

## Introduction

The human mouth with its diverse niches and ample supply of nutrients is undoubtedly conducive for the unrestricted formation of natural microbial biofilms. The oral microbial communities are some of the most complex microbial floras in the human body, consisting of more than 700 different bacterial species [1], [2]. Occurrence of disease results from disturbance of the equilibrium of this complex ecosystem, where population shifts lead to overrepresentation of pathogenic species which contribute to the onset and progression of the most common oral diseases, caries and periodontal disease [3]. Culture-independent molecular methods such as proteomics and 16S rRNA sequencing aiming to determine the bacterial diversity in the human oral cavity have demonstrated that in the supragingival plaque, *S. mutans* was the dominant species, with elevated levels of other streptococci including *S. sanguinis*, *S. mitis*, and *S. salivarius* in addition to lactobacilli and *Veillonella*. In contrast, the subgingival plaque was made up primarily of Gram-negative anaerobic bacteria such as *Fusobacterium nucleatum*, *Porphyromonas gingivalis*, and *Prevotella intermedia* which are known to be periodontal pathogens [3]–[5].

The dental tissues—enamel, dentin, and cementum—constitute the oral solid surfaces coated by a pellicle to which the microbial cells attach. The primary colonizers and secondary organisms stick to each other on the surface of teeth and generate a matrix of exopolysaccharide within which cells grow, forming a community with a collective physiology [6]. The resulting biofilm formed, known as dental plaque, subjects the teeth and gingival tissues to high concentrations of microbial metabolites which result in dental disease [2], [7]. The interactions between the various species in these mixed biofilms can be synergistic in that the presence of one microorganism generates a niche for other pathogenic microorganisms which can serve to facilitate the retention of organisms, an oral phenomenon known as coaggregation [3], [8]. The bacteria in the biofilm are always metabolically active which causes fluctuations in pH and loss of minerals from the tooth, ultimately resulting in dissolution of the dental hard tissues and formation of lesions known as dental caries [6], [9]. Interestingly, metabolic communications among oral bacteria may occur where the excretion of a metabolite by one organism is used as a nutrient by other organisms and breakdown of a substrate by enzymatic activity of one organism creates available substrates for different organisms [10], [11].

## Dental Caries

Dental caries or tooth decay is among the most prevalent human diseases, second only to the common cold [12]. Caries is a chronic disease that progresses slowly and is characterized by localized and irreversible destruction of the tooth [13], [14]. Despite scientific advancements in cariology in the past 150 years, dental caries remains a serious issue worldwide, particularly in children where it is the primary source of tooth loss. In the United States, 42% of children of ages between 2 to 11 have had dental caries in their primary teeth, and in the adult population, dental caries and periodontal diseases affect 60–90% of individuals worldwide [13]. People with disabilities and lower socioeconomic status suffer from the highest prevalence and pathogenicity of dental caries. Caries results from the complex interactions among the microbial species adhering to the tooth surface, with dietary, salivary, and genetic influences. The metabolic microbial interactions that take place in the dental biofilm result in acid production and extracellular glucan formation which promote microbial attachment to teeth [6], [12], [13]. Ninety percent of carious lesions occur in the pits and fissures of permanent posterior teeth and molar teeth as the biofilm tends to stagnate and mature in these areas which are relatively protected from mechanical wear by the tongue, cheeks, and tooth brushing [6]. The recognition of acid as the central etiological agent in dental caries initiated a search for the causative microorganisms in the oral microbiota, and in the early 1960s, the bacterial species *Streptococcus mutans* (*S. mutans*) became the main focus of caries research, assumed to be the specific cariogen [15].

## *Streptococcus mutans*: The Usual Suspect

*Streptococcus mutans* are Gram-positive bacteria that reside in the human mouth and, more specifically, in the multispecies biofilms on the surfaces of teeth [14]. *Streptococcus mutans* are major cariogenic organisms—the result of their ability to produce large quantities of glucans as well as acid, exceeding the salivary buffering capacities, which gives the bacteria an advantage to outcompete noncariogenic commensal species at low pH environments [9], [16]. This ability to survive in an acid environment by modulating sugar metabolic pathways coupled with irreversible binding to teeth is a key component to *S. mutans* pathogenesis. In the second stage of invasion, *S. mutans* coadhere or coaggregate with other microbial species, followed by proliferation and spread into other sites in the oral mucosa modulated by concerted action of genes and signaling molecules. In the final stage, the biofilm reaches a steady state which changes the equilibrium balance of the oral ecology; as a result, bacteria gain access into the deeper tissues and recesses in the gingival areas, ultimately causing dissolution of hydroxyapatite crystals in enamel and dentin which results in cavitation within the tooth [12], [17]. If not prevented, this cavitation provides an ecological niche where microorganisms form a protected biofilm, enabling caries to progress gradually [13]. Interestingly however, although it has been accepted for decades that *S. mutans* is the etiologic agent of dental caries, recent evidence indicates high prevalence for *S. mutans* in dental biofilms where the fungal pathogen *Candida albicans* (*C. albicans*) resides, suggesting that the interaction between these diverse species may mediate cariogenic development [18], [19].

## *Candida albicans:* Partner in Crime?

**Figure 1:**
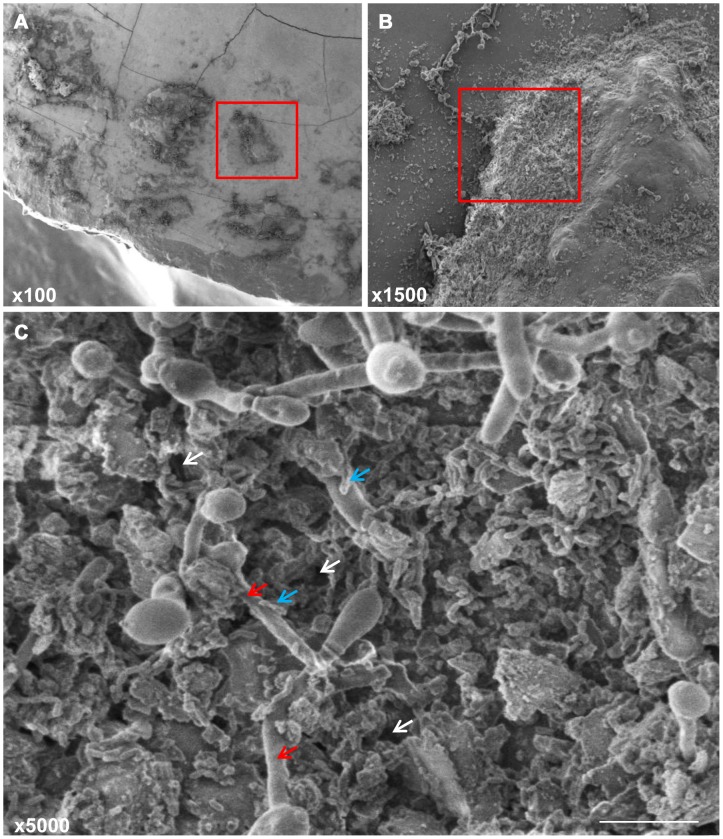
Scanning electron micrographs of mature mixed biofilms of *C. albicans* and *S. mutans* grown on extracted human teeth, demonstrating the tight coadherence between *C. albicans* hyphae (red arrows) and *S. mutans* cells (blue arrows). Microbial cells can be seen embedded in a matrix of extracellular polymeric substance with water channels (white arrows) through which liquid flows distributing nutrients and signaling molecules that facilitate communication between the cells. Bars 10 µm.

**Figure 2:**
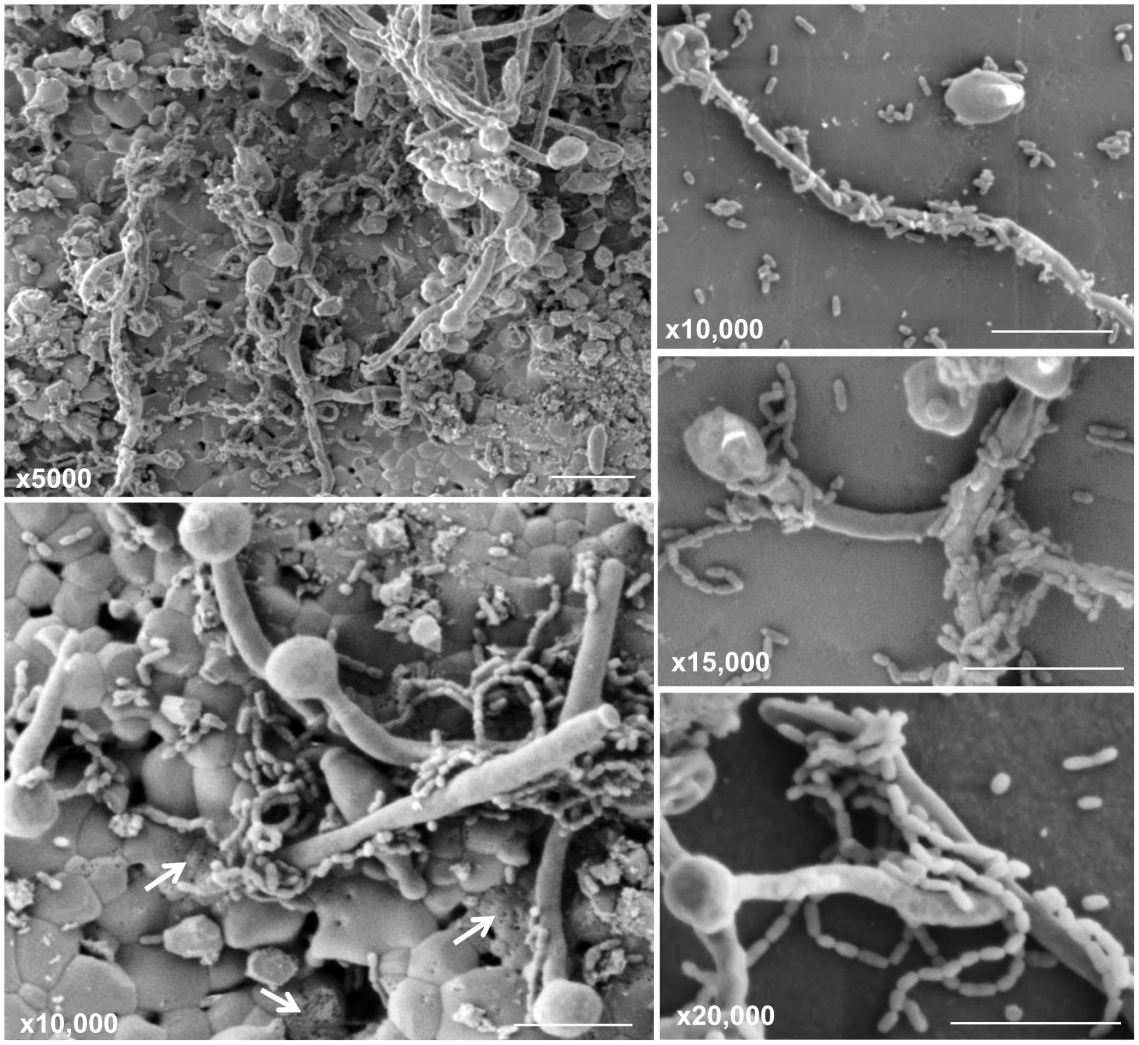
Scanning electron micrographs of mature mixed biofilms formed on discs of hydroxyapatite (a major component and essential ingredient of normal teeth), demonstrating the affinity of *S. mutans* to the hyphal elements of *C. albicans*. Bacterial cells are seen attached in chains as they adhere to and wrap around the hyphae. Perforations can be seen in the hydroxyapatite surface (white arrows), possibly the result of decalcification of the material due to the high acidic environment. Bars 10 µm.

*Candida albicans* is a commensal fungal species commonly colonizing human mucosal surfaces [20]. However, under conditions of immune dysfunction such as HIV infection, *C. albicans* can become opportunistic pathogens causing mucosal and disseminated infections. The ability of *C. albicans* to switch its morphology between yeast and hyphal forms contributes to its pathogenesis [20]. In the oral cavity, the coadhesion between *C. albicans* and oral bacteria is crucial for *C. albicans* colonization and persistence [21]. In addition to providing adhesion sites, the streptococci excrete lactate that can act as a carbon source for yeast growth, which in turn reduces oxygen tension to levels preferred by streptococci and provide growth stimulatory factors for the bacteria [21]. Increasing interest worldwide seems to focus on the role of *C. albicans* coaggregation with *S. mutans* during adherence to dental surfaces [18]. The hypothesis of the association between *S. mutans* and *C. albicans* is based on their mechanisms of virulence and biochemical characteristics as well as host factors that provide a buccal environment favoring the action of both microorganisms [17]–[19]. In fact, several *in vitro* studies have shown that *C. albicans* enhance the adherence of *S. mutans*, indicating a possible facilitation mechanism during their association where the yeast cells could be used by the bacteria as support for adherence [17], [19]. Scanning electron microscopic analysis of mixed species biofilms grown on human teeth and hydroxyapatite as substrata confirmed the strong coadherence between *C. albicans* and *S. mutans* to these surfaces and to each other, with *S. mutans* exhibiting high affinity to the *C. albicans* hyphae (Figures 1 and 2). More importantly, the yeast's potential to induce dental caries as a consequence of its pronounced ability to produce and tolerate acids was supported by a recent study by Klinke *et al.* [22], providing experimental evidence *in vivo* that *C. albicans* is capable of causing advanced occlusal caries in rats at a high rate. The findings from these *in vitro* and animal studies attributing a role for *C. albicans* in caries development and/or progression were solidified by data from a clinical study where the occurrence of caries in children was positively correlated with the frequency of oral candidal carriage [17]. Although still in its infancy, the research thus far strongly warrants that investigations on the microbiology of caries should include yeasts [22].

## Therapy and Challenges

As teeth loss in both children and adults has become a financial burden worldwide, understanding the homeostatic synchrony between the resident microflora, thereby preventing biofilm-induced caries and improving the quality of life, has become crucial. Although regular removal of biofilm on the tooth and the incorporation of fluoride in toothpaste has been the mainstay of dental caries prevention, these practices offer incomplete protection and may not effectively address the infectious character of the disease [6], [16]. Unfortunately, designing effective therapies to treat caries has been a major challenge, particularly in terms of oral drug delivery systems that can penetrate the biofilm networks of the target location [12], [16]. Antimicrobial peptides such as the histatins are considered promising agents due to their broad-spectrum antimicrobial properties [23], [24]. However, several questions about the use of these molecules as a tool for microbial control remain unclear. The recent search for more effective antimicrobials has explored the potential use of nanotechnology to improve the physical and chemical properties of drugs where nanosystem formulations incorporating different agents can improve stability and antimicrobial activity [25]. Other studies investigated the use of active inhibitors of enzymes that make up the exopolysaccharide matrix, thereby affecting the cariogenic biofilm assembly. Data generated from these studies demonstrated that in combination with fluoride, the new antibiofilm compounds successfully inactivated exopolysaccharide accumulation and matrix development [16]. However, although promising, these compounds are yet to be justified in *in vivo* studies to further evaluate their applicability paving the direction toward clinical applications.

## Conclusion and Future Directions

Microbial community interactions in health and caries pathogenesis are not well understood. It has been proposed that the translocation of oral bacteria to remote sites can lead to systemic diseases such as coronary artery disease [3]. Therefore, unraveling the basis of dental plaque development will ultimately contribute to both oral and overall health. To that end, using molecular techniques, current studies are focusing on identifying associations between oral bacteria and various oral and systemic diseases. Although *in vitro* studies can contribute to our understanding of the complex microbial associations and the dynamics of their interaction in the oral environment, the immunocompetence of the host and factors such as diet and oral hygiene play an important regulatory function. To better understand the impact of the interaction between *C. albicans* and *S. mutans* on caries development in a host, it is crucial to determine mechanistically precise details of adhesion and signaling under conditions of coexistence and to identify the molecular processes involved in the development of cariogenic biofilms in the host. By manipulation of adhesion interactions, it may be possible to develop new protocols to block adhesive reactions, impeding development of biofilm-related oral disease such as dental caries [9], [22], [26]. Therefore, the presence of *C. albicans* in the oral environment can now be considered an additional factor that needs to be taken into account in evaluating risks to caries [18]. To that end, future studies should focus on clinical studies and on designing animal model systems to study *in vivo*–grown polymicrobial biofilms, with the goal of developing novel therapeutic strategies to prevent dental caries through targeted actions.
